# One-stage tibial deformity correction and ankle arthrodesis for ankle osteoarthritis and tibial malalignment after low tibial osteotomy

**DOI:** 10.1016/j.ijscr.2021.106624

**Published:** 2021-11-25

**Authors:** Ichiro Tonogai, Koichi Sairyo

**Affiliations:** Department of Orthopedics, Institute of Biomedical Science, Tokushima University Graduate School, 3-18-15 Kuramoto, Tokushima City, Tokushima 770-8503, Japan

**Keywords:** Tibial osteotomy, Ankle arthrodesis, Anterolateral plate, Ankle osteoarthritis, Malalignment, Distal tibial osteotomy

## Abstract

**Introduction:**

There are no reports on one-stage corrective tibial opening wedge osteotomy and arthrodesis for osteoarthritis of the ankle and tibial malalignment after distal tibial osteotomy.

**Presentation of case:**

The patient was a 70-year-old woman who presented with complaints of ankle pain and lower limb deformity after tibial osteotomy performed for ankle arthritis 17–18 years earlier. Clinical examination revealed marked swelling around the ankle joint and pain and tenderness at the joint line. Imaging showed tibial malalignment and severe osteoarthritic changes in the ankle. The patient had valgus deformity of 21° and recurvatum deformity of 4°. In two months, she admitted to Department of Orthopedics at Tokushima University Hospital in Japan and we performed one-stage corrective tibial opening wedge osteotomy and ankle arthrodesis with an anterolateral plate through a lateral longitudinal incision. After removal of the previous implants, the remaining articular cartilage and osteophytes were removed from the tibial and talar surfaces. After debridement of the talar trochlea and tibial plateau, the center of rotation and angular deformity of the tibia was cut transversely and a 1-cm bone graft obtained from the removed fibula was inserted into the osteotomy site, which decreased the tibial malalignment. An anterolateral locking plate was inserted over the anterior and lateral sides of the tibia, and the ankle was fused using 2 cannulated screws.

**Discussion:**

The patient wore an above-knee splint for 6 weeks to avoid weight-bearing followed by gradual weightbearing with a brace thereafter. Osseous fusion was achieved after about 3.5 months. Radiographs obtained at the 2-year follow-up visit showed complete union of the tibia and talus. Full correction of valgus and recurvatum deformity was achieved, and the patient was able to perform daily activities with almost no pain.

**Conclusion:**

We reported a rare case of ankle osteoarthritis and tibial malalignment that was successfully treated with one-stage corrective tibial opening wedge osteotomy and ankle arthrodesis using an anterolateral plate via a transfibular approach.

## Highlights

• Ankle osteoarthritis and malalignment can be followed by osteotomy.
• One-stage ankle arthrodesis and deformity correction should be considered.
• Anterolateral plate was useful for arthrodesis and correction.

## 1 Introduction

Various surgical treatments have been described for osteoarthritis of the ankle [1]. Distal tibial osteotomy is a type of tibiotalar joint-preserving surgery and is indicated for asymmetric ankle osteoarthritis [2]. However, overcorrection can occur after distal tibial osteotomy [3]. Moreover, degenerative osteoarthritis of the tibiotalar joint continues in up to 25% of patients who undergo distal tibial osteotomy [4].

Rotational and angular deformities of the tibia can drive malalignment at the ankle joint, leading to shear stresses within the articular cartilage and altered contact pressures [5]. This asymmetric mechanical loading due to tibial deformity contributes to an increased risk of progression of osteoarthritis [6]. When ankle osteoarthritis is accompanied by limb deformity, it is reasonable to correct the more proximal deformity by first performing tibial osteotomy with an internal fixation method such as an intramedullary nail or external fixator, followed by total ankle arthroplasty in a staged manner to drive distal alignment [6], [7]. However, to our knowledge, there has been no description of a one-stage operative approach for osteoarthritis of the ankle with concomitant limb deformity after distal tibial osteotomy.

Here, we report a rare case of ankle osteoarthritis and tibial malalignment that was successfully treated by one-stage corrective tibial opening wedge osteotomy and ankle arthrodesis with an anterolateral plate via a transfibular approach. This has been reported in line with the SCARE criteria [8].

## 2 Presentation of case

The patient was a 70-year-old woman who was referred to our hospital approximately 17–18 years after undergoing distal tibial osteotomy for osteoarthritis of the left ankle at Mitoyo General Hospital (Mitoyo, Kagawa, Japan). It appeared that she had undergone lateral closing wedge osteotomy with a lateral plate but the details were unclear. Her left ankle pain did not improve after the initial surgery and gradually worsened. For further treatment, she was referred to Department of Orthopedics at Tokushima University Hospital (Tokushima, Japan). There was no relevant past medical history except for the above-mentioned operation and she was not on any medication.

**Fig. 1:**
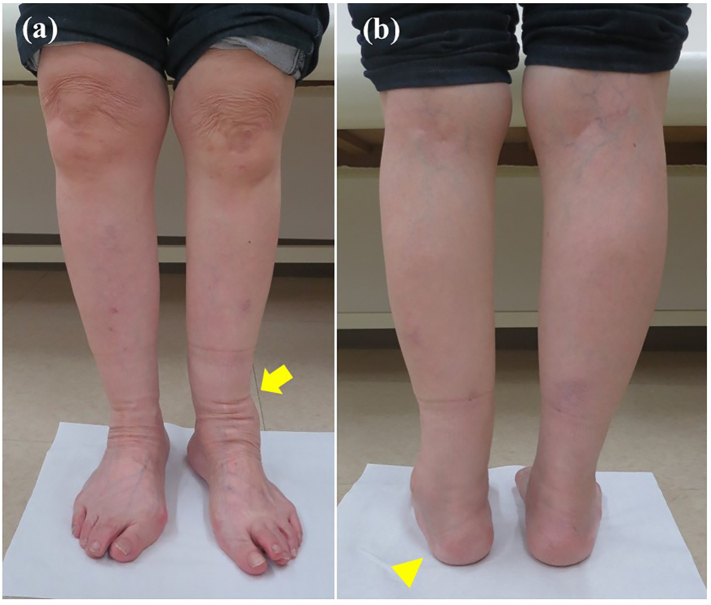
Standing clinical photographs showing (a) valgus deformity of the left lower leg on a frontal view (arrow) and (b) varus of the hindfoot on a rear view (arrowhead).

**Fig. 2:**
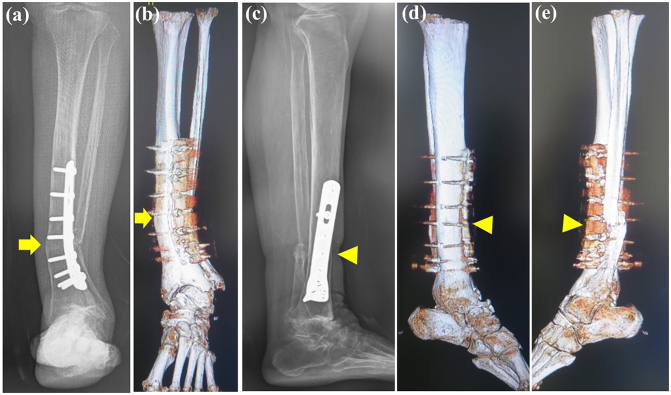
Valgus malalignment of the tibia as revealed by (a) a standing anteroposterior radiograph and (b) a 3-dimensional computed tomography scan (arrow). Recurvatum malalignment deformity of the tibia as revealed by (c) a standing lateral radiograph and (d) medial and (e) lateral 3-dimensional computed tomography scans (arrowhead).

**Fig. 3:**
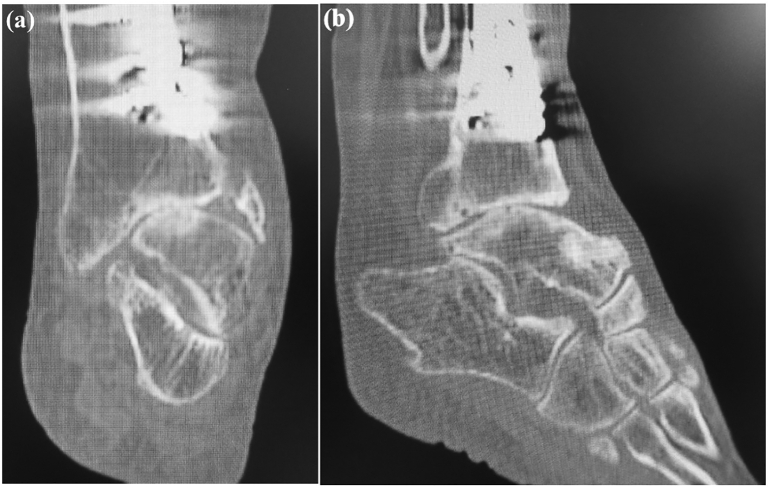
Varus lateral shift of the talus and severe osteoarthritic changes in the left ankle as revealed by (a) a coronal plain computed tomography scan. Severe ankle osteoarthritis, including sclerotic changes and cysts, as revealed by (b) a sagittal plain computed tomography scan.

On examination there was tenderness and swelling over the ankle joint. The lower leg showed valgus malalignment (Fig. 1a) and the hindfoot showed varus compensation (Fig. 1b) on standing. The range of motion at the ankle joint was less than that on the unaffected side, with dorsiflexion of 5° and plantar flexion of 15°. Preoperative measurements of the tibial axis on weight-bearing anteroposterior radiographs (Fig. 2a) and frontal 3-dimensional (3D) computed tomography (CT) scans (Fig. 2b) demonstrated valgus deformity of 21° and considerable lateral translation of the talus relative to the longitudinal axis of the proximal tibia. Weight-bearing lateral radiographs (Fig. 2c) and medial and lateral 3D CT scans (Fig. 2d, e) demonstrated 4° of recurvatum deformity. Coronal CT scans (Fig. 3a) of the ankle showed a varus talus and a valgus tibial plafond, and sagittal CT scans (Fig. 3b) showed severe osteoarthritis, with sclerotic changes, cysts, and osteophytes. We diagnosed osteoarthritis of the ankle with tibial malalignment after distal tibial osteotomy. We opted to remove the implants and perform a corrective tibial opening wedge osteotomy and ankle arthrodesis with an anterolateral plate via a transfibular approach two months after the first visit to us. Before surgery, the JSSF (Japanese Society for Surgery of the Foot) score was 16/100 (pain, 0/40; function, 16/50; alignment, 0/10).

**Fig. 4:**
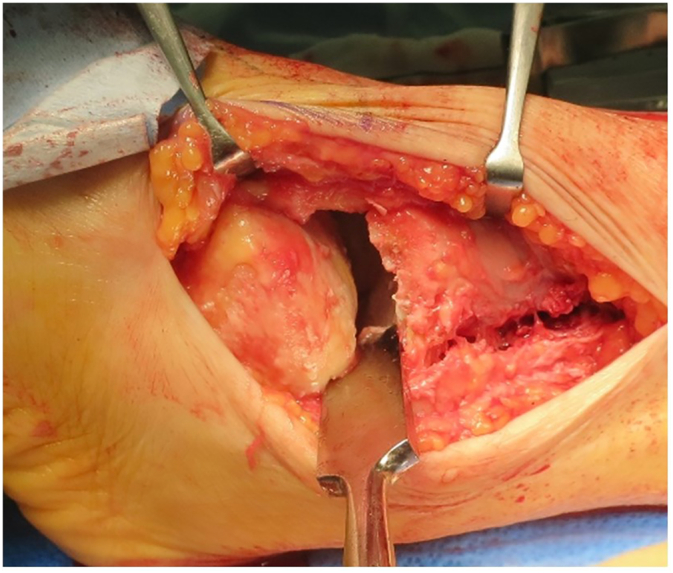
Intraoperative photograph via a transfibular approach. There is almost no cartilage and subchondral bone is exposed at the articular surface of the tibial plafond and talar trochlea. An osteophyte is present at the edge of the tibial plafond.

The surgery was performed by I.T. who graduated from the medical university in 2004 and was a foot and ankle surgeon. After induction of general anesthesia, the patient was positioned supine. A thigh tourniquet was applied. A 20 cm lateral linear incision was made along the fibula using the previous skin incision as a guide. Using a saw (Sagittal Saw Blades by Stryker; Kalamazoo, MI), osteotomy was performed to the distal fibula, which was then removed, exposing the ankle joint. The removed distal fibula was prepared for use as an autogenous 1-cm wedge bone graft. The implants were removed, but unfortunately ris of the tibial shaft occurred. Therefore, we had to select a longer plate than planned. The cartilage was almost completely gone and the subchondral bone was exposed at the articular surface of the tibial plafond and talar trochlea (Fig. 4). Using an osteotomy and a burr on the tibia and talus, we removed all the remaining articular cartilage and established beds of bleeding cancellous bone. Talotibial fusion was performed by inserting two cannulated screws (Hollyx; Numazu, Shizuika, Japan), each with a diameter of 6.5 mm. The first screw was inserted into the inferolateral aspect of the talus and directed proximally and medially and the second screw was inserted into the lateral side of the distal tibia and directed distally and medially. The center of rotation and angular deformity of the tibia was cut transversely by a bone saw and the interpositional 1-cm wedge bone graft harvested from the fibula was inserted. After reducing the valgus malalignment of the tibia and recurvatum deformity, fixation was established between the proximal tibia and talus using an anterolateral plate (LCP Anatomical Distal Tibia Plate; Depuy Synthes, West Chester, PA). The distal portion of the plate was placed at the anterior talus and the proximal portion along the lateral side of the tibia. The plate was confirmed to be in an appropriate position by fluoroscopy and then fixed with locking screws proximally and distally. Care was taken to ensure the subtalar joint was free of hardware intraoperatively by fluoroscopy. After closing the skin with sutures, long leg splinting was performed.

**Fig. 5:**
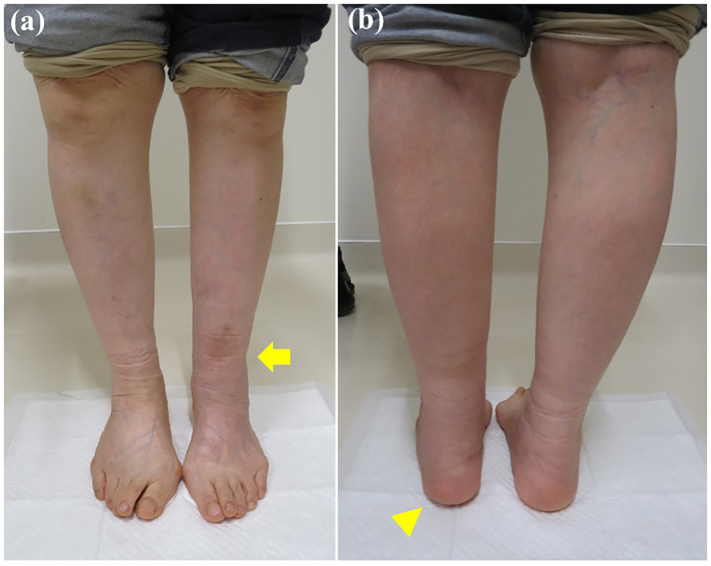
Standing clinical photographs obtained at the 2-year postoperative follow-up examination show improvement of the left-sided valgus deformity of the distal tibia and varus deformity of the hindfoot on (a) frontal view (arrow) and (b) rear view (arrowhead).

**Fig. 6:**
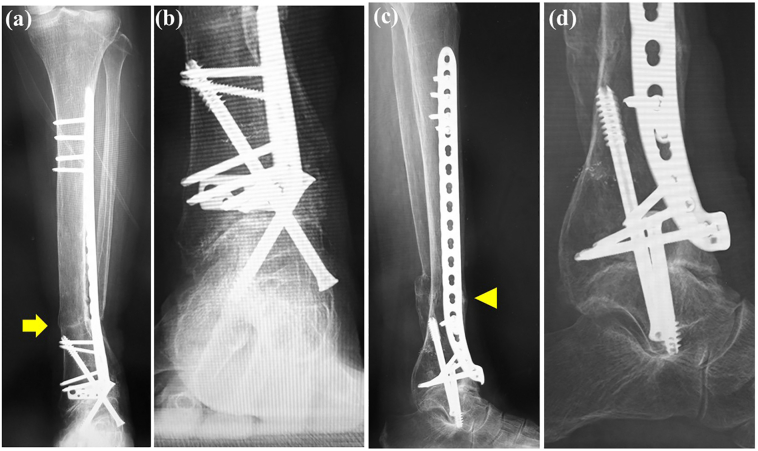
Standing anteroposterior radiographs of the (a) lower leg and (b) ankle obtained at the 2-year follow-up visit show correction of the valgus deformity of the tibia with complete bony consolidation at the sites of the corrective osteotomy and ankle fusion without dislocation or failure of the implant (arrow). Standing lateral radiographs of the (c) lower leg and (d) ankle obtained at the 2-year follow-up show correction of the recurvatum deformity of the tibia (arrowhead).

A non-weight-bearing above-knee splint that allowed range of motion exercises was applied. After 6 weeks of immobilization, the splint was removed and a long brace was attached to the ankle. Bony union of the ankle was achieved approximately 3.5 months after surgery. Gradual weight-bearing was permitted, and full weight-bearing was permitted 10 weeks after surgery. Clinical photographs obtained at the 2-year follow-up visit (Fig. 5a, b) indicated successful correction of the lower limb deformity. The valgus deformity seen on anteroposterior weight-bearing radiographs obtained preoperatively was corrected and within the normal range (Fig. 6a) and the talar angle tilt alignment was normal (Fig. 6b). Lateral weight-bearing radiographic views demonstrated correction of the recurvatum deformity to within normal range (Fig. 6c, d). Two years after surgery, the patient was pain-free with almost no limitation in her daily activities and an improvement in her JSSF score to 89/100 (pain, 40/40; function, 39/50; alignment, 10/10). The patient gave her permission for this case report to be published.

## 3 Discussion

There have been reports describing staged total ankle arthroplasty techniques for correction of limb deformity in patients with ankle osteoarthritis [6], [7]. However, this report describes a case of ankle osteoarthritis and tibial malalignment after distal tibial osteotomy in which one-stage corrective tibial osteotomy and ankle arthrodesis with an anterolateral plate via a transfibular approach was successful.

There are several reports on the clinical results of lateral closing wedge osteotomy for ankle osteoarthritis [9], [10]. Harstall et al. described 9 patients who experienced significant pain relief (with improvement in the pain score from 16 preoperatively to 30 postoperatively) and functional improvement (an increase in the American Orthopaedic Foot & Ankle Society hindfoot score from 48 preoperatively to 74 postoperatively) during a mean follow-up of 4.7 years [9]. Neumann et al. similarly reported that 21 of 27 patients were very satisfied with the results of surgery at the 6-month follow-up [10]. However, Colin et al. reported overcorrection in one of 43 patients who underwent lateral closing osteotomy [3]. Significant mechanical malalignment with subsequent displacement of the axis of motion of the ankle joint and altered load distribution and joint mechanics leads to increased stress, pain, articular cartilage damage, and subsequent degenerative disease [11]. Therefore, it is likely that overcorrection by lateral closing wedge distal tibial osteotomy caused the ankle osteoarthritis in the present case.

Arthrodesis remains the standard treatment for end-stage ankle arthritis because fusion typically provides a painless, plantigrade, and stable foot [12], [13]. Several fixation techniques and constructs have been described [14], [15], [16]. Currently, the most common fixation method is internal screw and/or plate fixation [17]. Several biomechanics studies have showed that plates achieve better stiffness and union rates than screws alone [18], [19], [20], [21], [22]. Given that we needed to achieve rigid fixation and to correct tibial malalignment in our patient, we opted for fixation using a plate with addition of screws in the tibiotalar joint.

Several investigators have used anterior [17], [23], [24], [25] or lateral [26], [27], [28] ankle joint bridging plates. However, the present report is the first to describe use of an anterolateral plate for arthrodesis of the ankle. Use of anterior plates has been associated with complications, including irritation of the extensor tendons and discomfort due to bulky implants, which necessitate removal of the hardware [29]. The distal portion of the plate was placed on the anterior side of the talus in our case, and there were no such complications, possibly because the anterior part of the plate was smaller than that of the plate usually used for ankle arthrodesis.

Open arthrodesis can be performed using several approaches [30]. Some surgeons prefer the transfibular approach described by Mann et al. in 1991 [31] over the anterior approach, probably because it provides a good view of both the anterior and posterior joint surfaces and poses less threat to neurovascular structures [32], [33], [34]. The soft tissue is thicker on the lateral side than on the anterior side, and complications such as wound infection, dehiscence, and delayed healing are less common with the transfibular approach than with the anterior approach. Therefore, we opted to use the transfibular approach. However, removal of the fibula has some drawbacks. The intact fibula provides an additional surface area for fusion, blocks valgus drift in cases of delayed union, and may serve as a guide for appropriate rotation and positioning of the talus within the mortise. Furthermore, preservation of the fibula maintains the native groove and restraints for the peroneal tendons. We wanted to preserve the fibula but needed to harvest a 1-cm wedge bone graft for corrective tibial opening wedge osteotomy. Moreover, in this case, the lateral portion of the distal tibia was rather bulky because there was a plate on the lateral side of the distal tibia. Skin closure would have been impossible in this case if the distal tibia was preserved. Therefore, we had to remove the distal fibula.

This report has some limitations. First, the follow-up duration of 2 years is relatively short. Ankle arthrodesis increases the contact stress on the talocalcaneal and calcaneocuboid joints. Patients who undergo ankle fusion for osteoarthritis go on to develop adjacent-joint arthritis after surgery. Therefore, further follow-up is necessary in this case to check for osteoarthritic changes in the joint adjacent to the tibiotalar joint. Second, placement of a long intramedullary nail in the subtalar joint via a plantar approach was an option. However, we consider it important to preserve an intact and flexible subtalar joint in a patient undergoing ankle arthrodesis in view of the study by Bai et al. demonstrating that patients lose 74% of sagittal range of motion, 70% of rotational range of motion, and 77% of valgus range of motion after ankle fusion [35]. Third, there was a slight amount of residual lateral shift of the talus relative to the longitudinal axis of the tibia. Nevertheless, the patient was satisfied with her treatment because she had a plantigrade foot and was pain-free when weight-bearing on her straight leg.

## 4 Conclusion

We have encountered a rare case of ankle osteoarthritis with tibial malalignment that we were able to treat successfully. This report suggests that one-stage corrective tibial osteotomy and ankle arthrodesis with an anterolateral plate via a transfibular approach is an effective procedure for this type of pathology.

## Declaration of competing interest

The Authors declare that there is no conflict of interest.
